# Prevalence of serum IgG antibodies against SARS-CoV-2 among clinic staff

**DOI:** 10.1371/journal.pone.0235417

**Published:** 2020-06-25

**Authors:** Simone B. Schmidt, Ludwig Grüter, Melanie Boltzmann, Jens D. Rollnik

**Affiliations:** 1 BDH-Clinic Hessisch Oldendorf, Institute for Neurorehabilitation Research, associated institute of Hannover Medical School, Hessisch Oldendorf, Germany; 2 Nordlab, Hameln, Germany; BronxCare Health System, Affiliated with Icahn School of Medicine at Mount Sinai, NY, UNITED STATES

## Abstract

The SARS-CoV-2 pandemic threatens health care providers and society. For planning of treatment capacities, it is of major importance to obtain reliable information on infection and fatality rates of the novel coronavirus. A German community study, the so-called Heinsberg study, found a 5-fold higher infection rate (and thus a remarkably lower fatality rate) than the officially reported cases suggest. We were interested to examine the SARS-CoV-2-IgG antibody status among clinic staff of a large neurological center in Northern Germany. Blood samples and questionnaires (demographic data, medical history) were collected pseudonymously. In total, 406 out of 525 (77.3%) of our employees participated in the study. The infection rate among the staff was as high as 2.7%. Including drop-outs (missing questionnaire but test result available), the infection rate was even higher (2.9%). Only 36% of the positively tested employees did suffer from flu-like symptoms in 2020. None of the nurses–having closest and longest contact to patients—were found to be positive. Despite the fact that the infection rate among clinic staff may not be directly compared to the situation in the surrounding county (due to different testing procedures), one might hypothesize that the infection rate could be more than 30-fold higher than the number of officially reported cases for the county of Hameln-Pyrmont. The high rate of IgG-positive, asymptomatic healthcare workers might help to overcome fears in daily work.

## Introduction

The family of Coronaviridea, within the order Nidovirales, contains several thousand different viruses (up to date 4189 complete genomes are sequenced [1]), which are sub-classified into the two subfamilies coronavirinae and torovirinae. Human corona viruses (HCoVs) were firstly detected 1965 by David A. J. Tyrrell and Bynoe [2] and may induce common colds, but also the severe acute respiratory syndrome (SARS) or the middle east respiratory syndrome (MERS). SARS uses to begin with flu-like symptoms, such as coughing, rhinitis, headaches, muscle and joint pains and diarrhea. A few days later, patients may suffer from fever and respiratory distress [3–4].

In the years 2002 and 2003 the first SARS-pandemic (SARS-CoV-1) occurred, about 8000 humans were infected and 9.6% died [5]. At the end of 2019, a new variant, the SARS-CoV-2, was detected in Wuhan/China for the first time. An infection by SARS-CoV-2 may induce COVID-19 (**Co**rona **vi**rus **d**isease 2019), which in most cases proceeds without- or only slight flu-like symptoms, however, in some cases with severe SARS-like symptoms. COVID-19 was declared as pandemic by the World Health Organization (WHO) on 11^th^ of March 2020 [6]. In total 3,349,786 confirmed COVID-19 infections and 238,628 deaths (fatality rate 7.1%) were reported worldwide (05/01/20) by the WHO [5]; in Germany, the Robert-Koch institute (RKI) published 164,807 confirmed cases (equaling a prevalence of confirmed cases of approximately 1.99 per thousand in the general population), leaving 6,996 dead (fatality rate 4.2%), on May 6^th^ 2020. A COVID-19 death is defined “as a death resulting from a clinically compatible illness in a probable or confirmed COVID-19 case, unless there is a clear alternative cause of death that cannot be related to COVID disease (e.g. trauma)” [7]. Prevalence as well as fatality rates of SARS-CoV-2 related infections has to be interpreted with caution because they are seriously distorted by case definition and detection, testing strategies, and reporting practice.

The so-called Heinsberg study focused on a small German community being a “hot spot” in the early phase of the pandemic [8]. The study enrolled 919 (out of 12,597) inhabitants of the village and obtained results from anti-SARS-CoV-2 IgG analyses in blood, polymerase chain reaction (PCR) testing for viral RNA in pharyngeal swabs and reported previous positive PCR tests. It turned out that 15.5% of the study participants were infected. Thus, infection rate was 5-fold higher than the number of officially reported cases for this community (3.1%). In addition, 22.2% of all infected individuals were asymptomatic; fatality rate was 0.36%, only.

The current SARS-CoV-2 pandemic is a major burden to healthcare providers such as hospitals, nursing homes and rehabilitation facilities. The lack of protective equipment such as particle filtering half masks (FFP2 or 3 masks) and the risk of poor patient care due to high sickness rates among medical staff is a tremendous challenge. Nurses as well as physicians are worried about the risk of infection with SARS-CoV-2 in contact with patients. Known immunity to the virus might help to overcome fears.

In the course of a SARS-CoV-2 infection, immunoglobulin G (IgG) antibodies may be detected after a median of 14 days (IQR 10–18 days) after onset of symptoms [9]. This B-cell response and the production of IgG antibodies play an important role in the neutralization of SARS-CoV-2 [10]. An IgG response therefore indicates an (possibly inapparent) infection as well as potential immunity. The immunity question is still a matter of debate, however, it may be concluded from experiments with primates that such an infection uses to induce immunity to SARS-CoV-2 [11]. Based on these study results and the experience with other CoV infections (SARS and MERS), the RKI currently suggests that those already infected could have a three-year immunity [12].

Due to the fact that COVID-19 occurs without symptoms in many cases, the prevalence resp. infection rate could be considerably higher than the number of confirmed cases. The present study intended to determine the prevalence of SARS-CoV-2 IgG antibodies among the clinical staff (immunity issue) and to get an estimate of the “dark figure” of unreported SARS-CoV-2 infections (prevalence/infection rate issue).

## Materials and methods

### Study design

The monocentric, prospective and observational study was designed and conducted at the BDH-Clinic Hessisch Oldendorf, a non-profit and specialized neurological center in Northern Germany. The study was accomplished according to the principles of good clinical practice of the Declaration of Helsinki. A positive vote was given by the ethics committee of Hannover Medical School to carry out the study (9016_BO_K_2020).

### Subjects and study procedures

After written, informed consent, we enrolled 406 employees (out of 525) of the BDH-Clinic Hessisch Oldendorf (participation rate 77.3%). The study was carried out between 20^th^ and 30^th^ of April 2020. Within this 10day period, blood samples were drawn and the presence of SARS-CoV-2 antibodies (in particular of the IgG class) in serum was analyzed. In addition, each subject completed a questionnaire. All data was collected in strictly pseudonymous form according to the study protocol.

### The BDH-Clinic Hessisch Oldendorf and the county of Hameln-Pyrmont

The BDH-Clinic offers 4 stroke unit-, 20 ICU (intensive care unit)-, 38 IMC (intermediate care)-, 51 normal ward- and 140 neurological rehabilitation beds. It is situated in a rural part of Lower Saxony, in the county of Hameln-Pyrmont, which has an area of 796 km^2^ and 148,559 inhabitants (data from 2019).

The first COVID-19 case in the county of Hameln-Pyrmont was reported on the 9^th^ of March 2020. Since then, 132 cases were confirmed and 10 humans died until the 5^th^ of May 2020 (fatality rate 7.6%). The prevalence of reported cases was as low as 0.889 per thousand. Until the 1^th^ of May 2020, a total of 4219 swab tests were carried out in the county of Hameln-Pyrmont (2.84% of the population).

In the clinic, swab tests did not yield positive results, neither among patients, nor among employees since occurrence of the pandemic situation. Only post-Covid-19-patients were admitted to the ICU for weaning from mechanical ventilation, transferred from other hospitals.

### Questionnaire

The questionnaire contained information about age group, gender, profession, flu-like infections this year, chronic underlying medical conditions, travels abroad in 2020 and pregnancy. When flu-like symptoms were reported, the duration of illness and details of symptoms (fever, cough, respiratory distress, muscle and joint pain, sore throat, headache, nausea, vomiting, rhinitis, diarrhea) were recorded. Among chronic underlying medical conditions, we asked the participants to submit information concerning cardiovascular-, respiratory-, hepatic- and cancerous diseases as well as diseases of the immune system and diabetes mellitus. For more information see Supporting Information (S1).

### Testing for SARS-CoV-2 IgG antibodies in serum

Venous blood samples were collected in VACUETTE^®^ CAT Serum Fast Separator (Greiner Bio-One GmbH, Kremsmünster, Austria). IgG antibodies against SARS-CoV-2 in serum were analyzed using an enzyme linked immunosorbent assay (ELISA) (Euroimmun Medizinische Labordiagnostika, Lübeck, Germany) carried out by an external service provider (Nordlab, Hameln, Germany). The ELISA test kit is a semi-quantitative in vitro assay for human antibodies of the IgG class against SARS-CoV-2 structural proteins in serum. In the first reaction step, diluted patient samples are incubated in the wells. In the case of positive samples, specific IgG antibodies (also IgA and IgM) will bind to the antigens. To detect the bound antibodies, a second incubation is carried out using an enzyme-labelled anti-human IgG (enzyme conjugate) catalyzing a color reaction. Results are semi-quantitative with a ratio (extinction of patient sample divided by extinction of calibrator) less than 0.8 interpreted as negative, and a ratio >1 as positive. The manufacturer reports a sensitivity of 94.6% in late cases (more than 10 days after onset of symptoms) with a specificity of 99.8%. There is a cross reactivity to SARS-CoV-1, only. The ELISA is the same product used in the Heinsberg study.

### Statistics

Statistical analysis was processed with SPSS software version 26.0 (SPSS Inc., Chicago, IL, USA). Statistical analyses were based on per protocol population, defined as subjects who gave a blood probe and completed the questionnaire. The results are presented as frequencies. Differences between groups were examined by χ^2^ test. P-values ≤0.05 were interpreted as statistically significant.

## Results

In total 406 subjects were enrolled in the study (77.3% participation rate). A complete questionnaire could be obtained from 385 subjects and collection blood sample was drawn in all 406 subjects. Thus, 21 subjects had to be excluded from data analysis and study population contains 385 subjects (77 men and 308 women) in different age classes and professional groups (Table 1).

**Table 1:** Demographical data of the study population.

	gender		
	male	female	total
age class			
18–29 years	4	51	55
30–49 years	37	117	154
50–64 years	32	138	170
>65 years	4	2	6
professional group			
nurse	15	139	155
physician	16	18	34
therapist	20	60	80
others	26	91	116
pregnancy	-	2	2
Total	77	308	385

193 subjects (50%) reported to suffer one (n = 158) or two (n = 35) infections since 1^th^ of January 2020. Most recent symptoms were headaches (27%), sore throat (32%), coughing (31%), rhinitis (24%) and muscle/ joint pain (20%). Only a few subjects reported fever (12%), diarrhea (n = 9%), nausea/vomiting (n = 7%) or respiratory distress (5%).

122 subjects had chronic medical conditions of the cardiovascular—(n = 69), respiratory—(n = 37), immune—(n = 18) and/or the hepatic system (n = 2) as well as cancer (n = 5) or diabetes mellitus (n = 11). A travel abroad was reported by 40 subjects.

The test for SARS-CoV-2 IgG antibodies revealed that 11 subjects had IgG antibodies against the virus. The prevalence among the staff was 2.9% (“per protocol” analysis). However, all subjects, who had to be excluded due to incomplete questionnaire, had a negative antibody test result, thus the prevalence was corrected to 2.7% (“intention-to-treat” analysis). The ratio of antibodies was between 1.1 and 6.5 (mean = 2.15, median = 1.6). Only four (36.4%) of the seropositive subjects had suffered symptoms: coughing (n = 4), fever (n = 2; 38.4°C and 38.5°C), headaches (n = 2), nausea/vomiting (n = 2), diarrhea (n = 2), muscle/joint pain (n = 1), sore throat (n = 2) and rhinitis (n = 1) (Table 2). The duration of symptoms within the subgroup of ill seropositive employees (n = 4) was between 4 and 21 days (mean = 11 days, median = 9 days). Seven of the seropositive subjects were between 30–49 years, three between 50–64 years and one subject between 18–29 years (Table 2). Three seropositive subjects are presently employed as therapists, three as physicians and five in other clinical sections (Table 2). None of the nurses were found to be seropositive. Two of the seropositive subjects have a cardiovascular condition and one subject has diabetes mellitus (Table 2). Only one subject had a history of a travel abroad (17 days in Europe/Baltic States) (Table 2).

**Table 2:** Comparison of the characteristics of seropositive-, seronegative- and undefined subjects.

	SARS-CoC-2 IgG antibody ratio groups		
	seronegative (<0.8) n = 371	undefined (0.8–1.0) n = 3	seropositive (>1.0) n = 11
gender			
male	72 (19%)	1 (33%)	4 (36%)
female	299 (81%)	2 (67%)	7 (64%)
age class			
18–29 years	53 (14%)	1 (33%)	1 (9%)
30–49 years	146 (39%)	1 (33%)	7 (64%)
50–64 years	166 (45%)	1 (33%)	3 (27%)
>65 years	6 (2%)	-	-
professional group			
nurse	154 (42%)	-	-
physician	31 (8%)	-	3 (27%)
therapist	75 (20%)	2 (67%)	3 (27%)
others	111 (30%)	1 (33%)	5 (46%)
subjects with infections	187 (50%)	2 (67%)	4 (36%)
time point of infection			
January 2020	34 (9%)	-	1 (25%)
February 2020	64 (17%)	2 (67%)	-
March 2020	46 (12%)	-	3 (75%)
April 2020	20 (5%)	-	-
missing data	23 (6%)	-	-
symptoms			
fever	41 (11%)	2 (67%)	2 (18%)
coughing	114 (31%)	2 (67%)	4 (36%)
respiratory distress	19 (5%)	1 (33%)	-
muscle/joint pain	73 (20%)	2 (67%)	1 (9%)
sore throat	119 (32%)	2 (67%)	2 (18%)
headaches	99 (26%)	2 (67%)	2 (18%)
nausea/vomiting	25 (7%)	-	2 (18%)
rhinitis	91 (24%)	1 (33%)	1 (9%)
diarrhea	32 (8%)	-	2 (18%)
medical condition			
cardiovascular disease	66 (18%)	1 (33%)	2 (18%)
respiratory disease	37 (10%)	-	-
weakened immune system	17 (5%)	1 (33%)	-
diabetes mellitus	10 (3%)	-	1 (9%)
hepatic disease	2 (1%)	-	-
cancerous diseases	5 (1%)	-	-
abroad stay	38 (10%)	1 (33%)	1 (9%)

Group comparisons of seropositive- and seronegative subjects showed that both groups did not differ in the percentages of subjects with medical conditions, symptoms in 2020 or reported symptoms during infections. In addition, no impact of age or gender could be detected.

## Discussion

In the rural and sparsely populated county of Hameln-Pyrmont, there is a low prevalence of reported COVID-19 cases (0.889 per thousand) compared to the general situation in Germany (about 1.99 per thousand). This low prevalence might not only be attributable to the population density, but also to a relatively small number of performed PCR swab tests (2.8% of the general population).

A community study, the so-called Heinsberg study, already found a high number of unreported, unknown cases and demonstrated that 15.5% of the study participants were infected. This was found to be 5-fold higher than the number of officially reported cases for this “hot spot” community [8].

Nevertheless, it is surprising that 2.7%/2.9% were found to be seropositive among the clinical staff, while only 0.889 per thousand cases were reported in the surrounding county. Only four of the seropositive employees suffered from symptoms, none of them had a swab test before to check for an acute infection with SARS-CoV-2. The detection of antibodies against SARS-CoV-2 via ELISA is highly sensitive (94.6%) and specific (99.8%). The manufacturer of the ELISA test reports that antibodies may be detected as early as 10 days after symptom onset in most cases, while a few cases exhibit a highly delayed antibody production (more than four weeks). This finding might be caused by an individual host immune response. This might have occurred in our study sample, too, resulting in some false negative test results. In this case, the infection rate could even be higher than calculated and thus does not compromise main conclusions of the present study.

Due to the study design, the results from this study are not representative for a general, ageing population and the situation in the surrounding county. Our study focused on younger, working people, but it has to be pointed out that the Heinsberg study did not find age to be associated with the infection rate [8]. In our study, age did not play a role, either. On the other hand, it is reasonable to assume that healthcare professionals have a higher risk to be infected during a pandemic because they are more likely to have contact to infectious patients. However, there was no SARS-CoV-2 outbreak in our hospital; nor patients, neither employee have been found to be positive in PCR swab tests. Upon admission, all patients were tested for clinical symptoms. If suspicious (fever, respiratory symptoms), contact precautions were carried out until a negative PCR result was obtained.

Apparently, the situation in a neurological clinic may not be compared to a general hospital with much more patients primarily seeking help due to respiratory symptoms. In addition, all our employees are constantly carrying FFP1 masks for their protection when in contact with patients or co-workers since the 7^th^ of April 2020.

A major finding is that none of the nurses turned out to be seropositive while nurses use to have the closest and longest contact to patients. These specific conditions put the authors into a position to assume that the seropositive employees might have been infected outside the clinic and not by patients. Nevertheless, the infection rate among healthcare workers may not directly compared to the situation in the surrounding county. There are huge differences in analysis procedures (PCR swab test in sick patients in the general population vs. antibody test in healthy clinic staff) as well as different time periods. These limitations hamper a direct comparison. However, one might hypothesize that the infection rate could be more than 30-fold higher than the number of officially reported cases for the county of Hameln-Pyrmont. A higher infection rate among healthcare workers might be explained by more human contacts than in home-working employees. In addition, the higher infection rate observed in this study would have a major impact on the fatality rate which is calculated on the basis of the infection rate. The alarming fatality rate of 7.6% in our county would have to be re-adjusted to a far lower value like the authors of the Heinsberg study have suggested.

An infection rate of 2.7% (resp. 2.9%) indicates that there is a considerably higher than assumed number of (potentially) immune employees. The knowledge of their antibody status might help to overcome fears. In addition, these staff members could be preferred to treat infectious COVID-19 patients because they have a much lower risk to be affected by this contagious virus than persons found to be negative in IgG antibody testing.

## Supporting information

S1 Questionaire Used questionnaire for the study participants. (PDF)

## References

[1] Coronaviridae Taxonomy Browser, https://www.viprbrc.org/brc/home.spg?decorator=corona (Accessed on 4 May 2020)

[2] TYRRELL DA and BYNOE ML. CULTIVATION OF A NOVEL TYPE OF COMMON-COLD VIRUS IN ORGAN CULTURES. *Br Med J* 1965; 1: 1467–1470. 10.1136/bmj.1.5448.1467 14288084 PMC2166670

[3] Peiris, Joseph S M, Yuen KY, Osterhaus, Albert D M E and Stöhr K. The severe acute respiratory syndrome. *N Engl J Med* 2003; 349: 2431–2441. 10.1056/NEJMra032498 14681510

[4] Donnelly CA, Ghani AC, Leung GM, et al Epidemiological determinants of spread of causal agent of severe acute respiratory syndrome in Hong Kong. *Lancet* 2003; 361: 1761–1766. 10.1016/S0140-6736(03)13410-1 12781533 PMC7112380

[5] Mahase E. Covid-19: WHO declares pandemic because of "alarming levels" of spread, severity, and inaction. *BMJ* 2020; 368: m1036 10.1136/bmj.m1036 32165426

[6] WHO (World Health Organization). Summary of probable SARS cases with onset of illness from 1 November 2002 to 31 July. http://www.who.int/csr/sars/country/table2004_04_21/en/index.html (Accessed on 4 May 2020).

[7] WHO (World Health Organization). Coronavirus disease (COVID-19) Situation Report– 104. https://www.who.int/docs/default-source/coronaviruse/situation-reports/20200503-covid-19-sitrep-104.pdf (Accessed on 4 May 2020)

[8] Streeck H, Schulte B, Kümmerer BM, Richter E, Höller T et al. (2020). Infection fatality rate of SARS-CoV-2 infection in a German comminity with a super-spreading event. https://www.ukbonn.de/C12582D3002FD21D/vwLookupDownloads/Streeck_et_al_Infection_fatality_rate_of_SARS_CoV_2_infection2.pdf/$FILE/Streeck_et_al_Infection_fatality_rate_of_SARS_CoV_2_infection2.pdf (Accessed on 6 May 2020) 10.1038/s41467-020-19509-y PMC7672059 33203887

[9] Guo L, Ren L, Yang S, et al Profiling Early Humoral Response to Diagnose Novel Coronavirus Disease (COVID-19). *Clin Infect Dis* 2020 10.1093/cid/ciaa310 32198501 PMC7184472

[10] Zhou G and Zhao Q. Perspectives on therapeutic neutralizing antibodies against the Novel Coronavirus SARS-CoV-2. *Int J Biol Sci* 2020; 16: 1718–1723. 10.7150/ijbs.45123 32226289 PMC7098029

[11] Bao L, Deng W, Gao H, Xiao C, Liu J, Xue J, et al. Reinfection could not occur in SARS-CoV-2 infected rhesus macaques. bioRxiv. 2020:2020.03.13.990226. https://www.biorxiv.org/content/10.1101/2020.03.13.990226v1 (Accessed on 4 May 2020)

[12] SARS-CoV-2 Steckbrief zur Coronavirus-Krankheit-2019 (COVID-19). https://www.rki.de/DE/Content/InfAZ/N/Neuartiges_Coronavirus/Steckbrief.html#doc13776792bodyText23 (Accessed on 4 May 2020)

