# Supplementary material for: Prevalence of serum IgG antibodies against SARS-CoV-2 among clinic staff
Source: PLoS One. 2020 Jun 25;15(6):e0235417. doi: 10.1371/journal.pone.0235417 (PMC7316280; doi:10.1371/journal.pone.0235417)
Supplement: S1 Questionaire — (PDF) [file pone.0235417.s001.pdf]

S1: Used questionnaire for the study participants.

Original language version (German)

Pilotstudie zur Prävalenz der Immunität gegen SARS-CoV-2 im medizinischen Personal einer Klinik

Altersgruppe:

☐ Kinder und Jugendliche zwischen sechs und 17 Jahren,
☐ Erwachsene zwischen 18 und 29 Jahren,
☐ Erwachsene zwischen 30 und 49 Jahren,
☐ Erwachsene zwischen 50 und 64 Jahren,
☐ Erwachsene im Alter von 65 Jahren und älter

Geschlecht: ☐ männlich
☐ weiblich
☐ divers

Berufsgruppe ☐ Pflegedienst
☐ Arzt
☐ Therapeut
☐ andere Berufsgruppe (z. B. Verwaltung)

Haben Sie im Jahr 2020 Infekte durchgemacht?

☐ **Nein**, ich war in den letzten Monaten (Januar bis jetzt) überhaupt nicht krank.

☐ **Ja**, ich habe folgende Infekte durchgemacht:

Datum der Infekte				
Dauer der Krankheit (Tage)				
Symptome (bitte ankreuzen):				
Fieber (wenn ja: wie hoch in °C)				
Husten				
Atemnot				
Muskel-/ Gelenkschmerzen				
Halsschmerzen				
Kopfschmerzen				
Übelkeit/Erbrechen				
Verstopfte Nase				
Durchfall				
Andere Beschwerden (bitte aufschreiben)				

S1: Used questionnaire for the study participants.

Bei mir liegen folgende Vorerkrankungen vor (bitte ankreuzen, wenn vorliegend):

☐ Herz-Kreislauf-System (z. B. koronare Herzerkrankung, Bluthochdruck): ....

....

☐ Lunge (z. B. Asthma, chronische Bronchitis): ....

☐ chronische Lebererkrankungen: ....

☐ Diabetes mellitus

☐ Krebserkrankung

☐ geschwächtes Immunsystem (z. B. aufgrund einer Erkrankung, die mit einer Immunschwäche einhergeht oder durch Einnahme von Medikamenten, die die Immunabwehr schwächen, wie z. B. Cortison): ....

....

Haben Sie in den vergangenen drei Monaten Auslandsaufenthalte gehabt?

☐ nein
☐ ja: vom (Datum) .... bis ....

in ....

Für Frauen: Sind Sie schwanger?

☐ ja, ... Woche (SSW)
☐ nein

S1: Used questionnaire for the study participants.

<u>English version</u>

Pilot study of the prevalence of serum IgG antibodies against SARS-CoV-2 among clinic staff

Age group:

☐ Children and teenager between six and 17 years,
☐ Adults between 18 and 29 years,
☐ Adults between 30 and 49 years,
☐ Adults between 50 and 64 years,
☐ Adults of 65 years and older.

Gender: ☐ male
☐ female
☐ divers

Professional group ☐ nurse
☐ physician
☐ therapist
☐ others (i.e. administration)

Did you go through infections in 2020?

☐ **<u>No</u>**, I did not feel sick between first of January until now.

☐ **<u>Yes</u>**, I went through the following infections:

Date of infection:				
Duration illness (days)				
Symptom<br>(please tick off):				
Fever (if yes, which temperature in °C)				
Coughing				
Respiratory disstress				
Muscle/joint pain				
Sore throat				
Headaches				
Nausea/vomiting				
Rhinitis				
Diarrhea				
Other complaints (please notice)				

S1: Used questionnaire for the study participants.

I have the following pre-existing conditions (please tick off if present):

☐ Cardiovascular system (i. e. coronary heart disease, high blood pressure): ....

....

☐ Respiratory system (i. e. Asthma, chronic bronchitis): ....

☐ Chronic liver diseases: ....

☐ Diabetes mellitus

☐ Cancer

☐ Weakened immune system (i. e. due to a condition that is associated with an immune deficiency or by taking drugs that weaken the immune system, such as cortisone): ....

....

Have you been abroad in the past three months?

☐ No
☐ Yes: from (Date) .... to ....

in ....

For women: Are you pregnant?

☐ Yes, ... …… (week of pregnancy)
☐ No
